# Supplementary material for: Ultrasensitive detection of local acoustic vibrations at room temperature by plasmon-enhanced single-molecule fluorescence
Source: Nat Commun. 2022 Jun 9;13:3330. doi: 10.1038/s41467-022-30955-8 (PMC9184529; doi:10.1038/s41467-022-30955-8)
Supplement: Supplementary file 1 — Supplementary Information [file 41467_2022_30955_MOESM1_ESM.pdf]

Supplementary Information for

Ultrasensitive detection of local acoustic vibrations at room temperature by plasmon-enhanced single-molecule fluorescence

Mingcai Xie[1], Hanyu Liu[1], Sushu Wan[1], Xuxing Lu[1,2], Daocheng Hong[1], Yu Du[1], Weiqing Yang[1], Zhihong Wei[1], Susu Fang[1], Chen-Lei Tao[3], Dan Xu[3], Boyang Wang[4], Siyu Lu[4], Xue-Jun Wu[3], Weigao Xu[1], Michel Orrit[2,*], and Yuxi Tian[1,*]

[1]Key Laboratory of Mesoscopic Chemistry of MOE, School of Chemistry and Chemical Engineering, Nanjing University, Nanjing 210023, China

[2]Huygens-Kamerlingh Onnes Laboratory, Leiden University, 2300 RA Leiden, The Netherlands

[3]State Key Laboratory of Coordination Chemistry, School of Chemistry and Chemical Engineering, Nanjing University, Nanjing 210023, China

[4]Green Catalysis Center, and College of Chemistry, Zhengzhou University, Zhengzhou 450001, China

E-mail address: orrit@physics.leidenuniv.nl; tyx@nju.edu.cn

Supplementary Note 1: OPTICAL SETUP

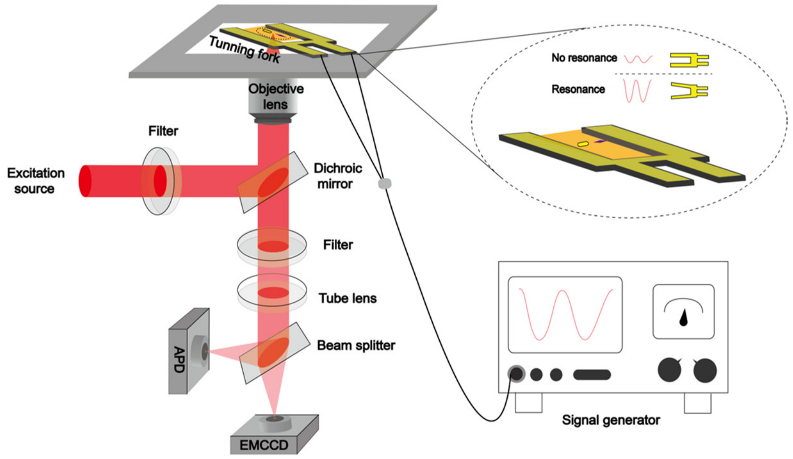

Supplementary Figure 1: Schematic diagram of our home-built wide-field fluorescence microscope. A 633 nm laser was used as the excitation light source and the fluorescence from a single CV molecule was collected and imaged on an APD and an EMCCD, respectively. When an electric voltage was applied to the tuning fork using a signal generator, the amplitude of the sine wave observed in an oscilloscope became much bigger and sharper suddenly. The signal generator with variable voltages ranging from 0 to 20 V could provide different extent amplitudes to generate different deformations of the tuning fork at its resonant frequency, which induces periodic distance variations between the molecule and the gold nanorod. The APD detected the variations of fluorescence number of counts emitted by a single molecule when its distance to the gold nanorod changed periodically. The distance variations lead to variations in the enhancement of the molecular fluorescence by the gold nanorod.

Supplementary Note 2: PHOTOSTABILITY CHARACTERIZATION AT SINGLE-MOLECULE LEVEL

Good photostability is a basic requirement for molecules as fluorescent probes. Thus, we checked the photostability of CV molecules at the single-molecule level. We took on/off-time histograms as characterizations for single-molecule bleaching. In Supplementary Figures 2a–d, we provide detailed calculation methods of the on-time, off-time, total on-time and total off-time. Supplementary Figure 2a presents a schematic diagram of single-molecule fluorescence intensity traces with two-state blinking. The number labelled on the blinking stages represents the duration of the on-state or off-state. We can easily count the present frequency of different on/off-time in the blinking stages. For example, we obtained 5 times for the on-time duration of 3s by simple counting. Similarly, we calculated different on-time and off-time histograms as shown in Supplementary Figures 2b and 2c, respectively. In

addition, we calculate the dwell time of a molecule in the fluorescent state and dark state to investigate the bleaching behavior of a molecule under continuous light excitation. As indicated in Supplementary Figure 2d, we use the total on-time and total off-time to characterize the dwell time of a molecule in the fluorescent state and dark state by summing up on-times and off-times from single-molecule fluorescence intensity traces in Supplementary Figure 2a, respectively.

We tracked the fluorescence signal of single CV molecules under continuous light excitation for 10 min, as presented in Supplementary Figures 2e and 2f, although most events mainly occurred in the high off-time range, there are still many events occurred in the high on-time range. The photostability of CV molecules at single-molecule level is relatively good. Besides, we also calculated the total on/off-time of individual CV molecules, as shown in Supplementary Figure 1b of the main text and Supplementary Figure 2g. Although most CV molecules can be bleached within 10 min, many molecules still can survive for a long time. To obtain stable and reliable acoustic signals, we automatically selected the surviving molecules for the acoustic detection measurements.

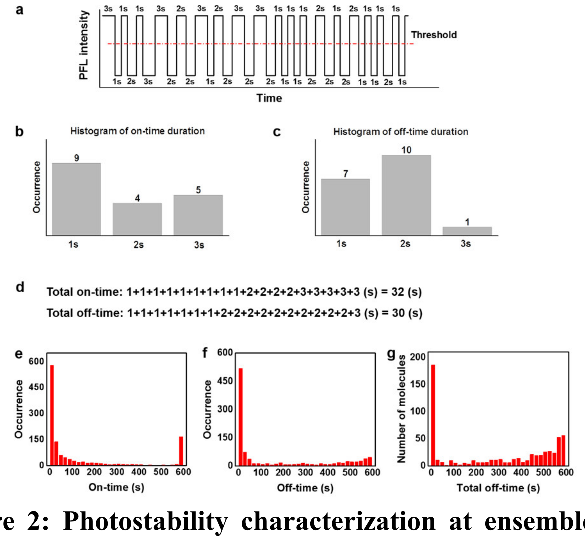

Supplementary Figure 2: Photostability characterization at ensemble and single-molecule levels. a–d The schematic illustration for statistics and calculation: **a** the fluorescence intensity traces in individual molecules; **b** on-time calculation; **c** off-time calculation; **d** total on-time and off-time calculation. **e–f** Statistics of on/off-state duration of single CV molecules under light irradiation for 10 min. Although most events occurred in high-off-time range, there are still many events occurring in the high-on-time range. **g** Statistics of total on-state duration time of single CV molecules under continuous light irradiation for 10 min. Although most CV molecules can be bleached within 10 min, many molecules still can survive for a long time.

Supplementary Note 3: GEOMETRIC SIZE CHARACTERIZATION OF GOLD NANORODS

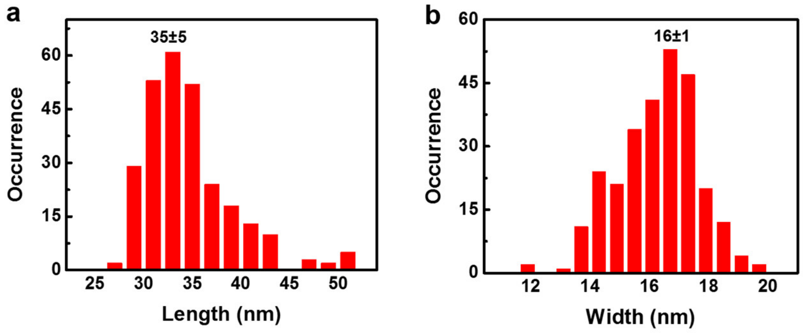

Supplementary Figure 3: Characteristic distribution of the geometric size of gold nanorods. a Length distribution. The average length is 35±5 nm. **b** Width distribution. The average width is 16±1 nm.

Supplementary Note 4: CHARACTERIZATION OF THE TUNING FORK

To characterize the tuning fork without and with sample, we scanned the driving frequencies around the central resonant frequency of the tuning fork without (from 32.735 to 32.781 kHz) and with (from 32.650 to 32.779 kHz) sample using a signal generator with a driving voltage of 5 V by a confocal microscope, as shown in Supplementary Figure 4a. In details, we illuminated one arm of the tuning fork with a laser focus spot of 532 nm and half of the laser focus spot was reflected and the other half was transmitted. The arm of the tuning fork would vibrate periodically when the driving frequency was at its resonant frequency. Then, the transmission intensity would be changed and we extracted the variation by a lock-in amplifier. In this way, we can obtain the resonant spectra of the tuning fork without and with sample as shown in Supplementary Figures 4b and 4c which shows a central resonant frequency of 32.758 and 32.714 kHz, respectively. The Q factor decreases from 5,000 to 2,000 after the sample is attached due to additional dissipation. For the following acoustic detection measurements, we fixed the frequency of the tuning fork to be on resonance to generate the strongest acoustic wave. It is worth noting that the resonant frequency of the tuning fork slightly varies from sample to sample. Thus, we have to characterize the resonant spectrum of each sample before measurements.

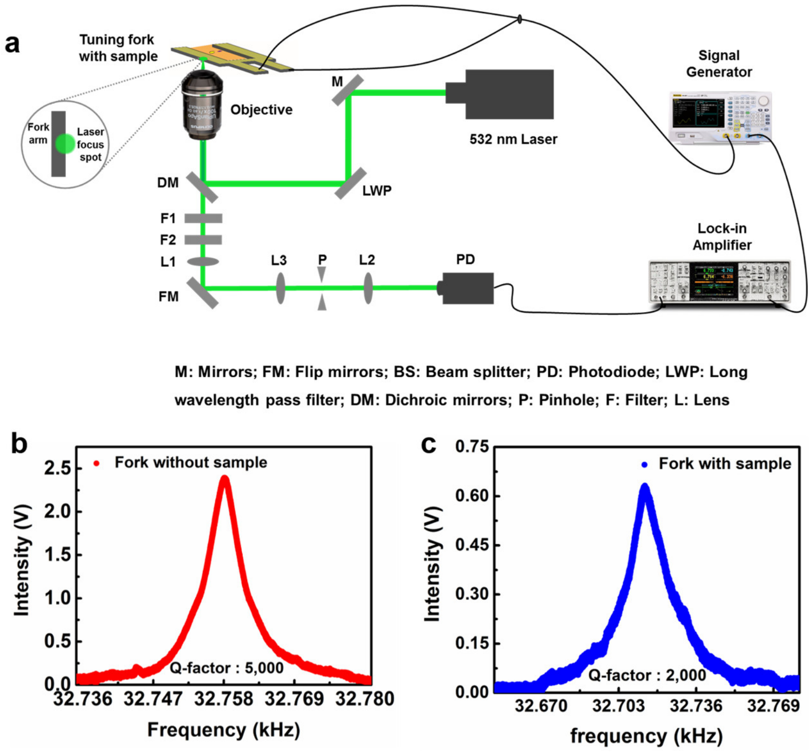

Supplementary Figure 4: Characterization of the tuning fork. a Schematic diagram of our confocal microscope. **b** The resonant spectrum of the tuning fork without sample. The central resonant frequency is 32.758 kHz with Q factor of 5,000. **c** The resonant spectrum of the tuning fork with sample. The central resonant frequency is 32.714 kHz with Q factor of 2,000. The illumination light source is 532 nm and the driving voltage is 5 V.

Supplementary Note 5: CONFIRMATION OF THE FLUORESCENCE ENHANCEMENT BY THE GOLD NANOROD

In order to confirm that the fluorescence of a single CV molecule is truly enhanced by a gold nanorod, we compared the fluorescence image and the corresponding scattering image at the same position as shown in Supplementary Figure 5. We can find corresponding gold nanorods (bright spots in the scattering image of Supplementary Figure 5b) for all the emissive CV molecules (bright spots in the fluorescence image of Supplementary Figure 5a), which can confirm that only the molecules staying close to the gold nanorod can be enhanced and detected. Furthermore, we also measured the lifetime of CV molecules without addition of gold nanorods embedded in the PMMA film at ensemble level, which is ~0.86 ns as shown in Supplementary Figure 5c. For comparison, we measured the lifetime of the bright spot of CV fluorescence enhanced by the gold nanorod around, which is ~0.02 ns as shown in Supplementary Figure 5d

and far shorter than that of CV molecules without gold nanorods as measured on an ensemble. This result further confirms that the bright spots of fluorescence are enhanced by gold nanorods, because the collective enhancement of the radiative decay rates and additional nonradiative decay rates leads to a decreased lifetime.

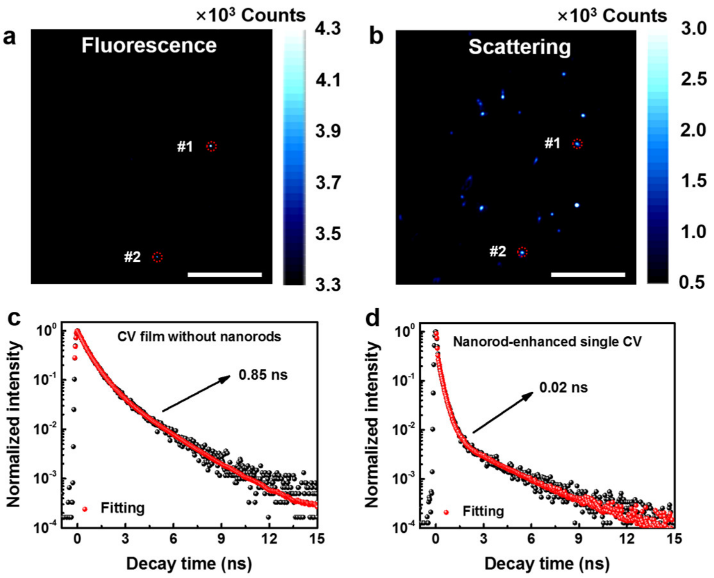

Supplementary Figure 5: Characterization of the molecular fluorescence enhancement by the gold nanorod. a Fluorescence image of CV molecules; Scale bar: 20 μm. **b** Scattering image of gold nanorods at the same area as the fluorescence image; Scale bar: 20 μm. All the bright spots in the fluorescence image are associated to bright spots at the same coordinates in the scattering image. **c** The lifetime of CV molecules without gold nanorods embedded in the PMMA film measured on an ensemble. The measured lifetime is ~0.86 ns. **d** The lifetime of the bright spot of CV fluorescence enhanced by the gold nanorod in its vicinity. The measured lifetime is ~0.02 ns, which is far less than that of CV molecules without gold nanorod, further confirming that the bright spot of fluorescence is truly enhanced by the nearby gold nanorod.

We also estimated the probability to find a molecule in the near field of a gold nanorod. The estimation can be divided into three steps. First, we calculated the number of CV molecules in the photoexcitation field. The photoexcitation field's volume was calculated to be 1.5×10^{-7} cm^3 according to the excitation area of 7.5×10^{-5} cm^2 and the film thickness of 2.0×10^{-3} cm. Then, the number of CV molecules in the photoexcitation field was determined to be ~5.0×10^{6} according to the concentration of 5.0×10^{-8} M of molecules in the fabricated film. Second, we calculated the number of gold nanorods in the photoexcitation field. The concentration of gold nanorods in the fabricated film was 2.0×10^{-11} M, so the number of gold nanorods in the

photoexcitation field was calculated to be ~2.0×10^3 according to the photoexcitation field's volume of 1.5×10^{-7} cm^3. Unfortunately, the thickness of the fabricated film in our experiment is very inhomogeneous due to the high boiling point of the DMSO solvent, which is hard to evaporate quickly during spin-casting. However, for our experiments, we selected thick areas for investigation in order to scratch the film easily. In that case, the number of gold nanorods in the thick area may be much higher than that in the other thin areas. Therefore, we roughly estimated the number of gold nanorods in the thick area to 10 times higher than the average number. Thus, the number of gold nanorods in the photoexcitation field is 2.0×10^4. Last, we calculated the probability to find a molecule near a gold nanorod in the photoexcitation field. We only need to calculate the ratio between the total volume of these gold nanorods and the photoexcitation field volume since the size of a molecule is ~2 nm and can be neglected. The volume of a single gold nanorod was calculated to be about ~8×10^{-18}cm^3 according to the size of 35×16 nm and the molecule-nanorod distance of 5 nm. Therefore, the probability of a single molecule being located near a gold nanorod is ~1.0×10^{-6} according to the number of 2.0×10^4 and the photoexcitation field's volume of 1.5×10^{-7} cm^3. Since we have 5.0×10^6 molecules in the excitation area, we would be able to detect 5 molecules which is reasonably in agreement with our experimental results.

Supplementary Note 6: LINEAR DEPENDENCE OF FFT PEAK ON THE DRIVING VOLTAGE

To be more reliable and convincing, we also investigated the dependence of FFT peaks on the driving voltage in another two molecules in response to the acoustic wave. Supplementary Figures 6a and 6c show the respective fast Fourier transforms (FFT) of the fluorescence intensity traces of single molecules #1 and #2, which indicate sensitive responses to the acoustic wave. The FFT amplitude has been calibrated in pico-meters corresponding to the variation of the distance between the single molecule and the gold nanorod. Notably, the calibration is different for

different molecules. Then, we plotted the respective FFT peaks of single molecules #1 and #2 as functions of the driving voltage as shown in Supplementary Figures 6b and 6d. We found that the FFT peak of molecule #1 shows excellently linear dependence on the driving voltage. However, the molecule #2 exhibits a small deviation from the linear fitting, which may be due to environmental fluctuations. As a whole, the FFT peaks in both molecules vary linearly with the driving voltage.

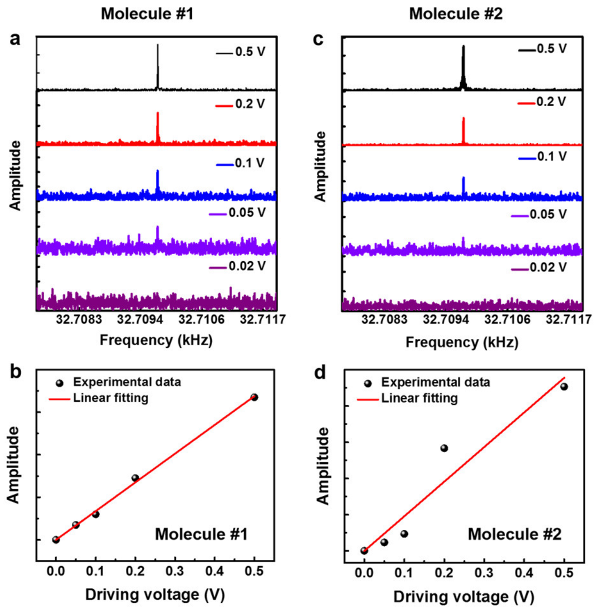

Supplementary Figure 6: Characterizations of linear dependence of FFT peaks on the driving voltage. a, **c** Respective fast Fourier transform (FFT) of the fluorescence intensity traces of single molecules #1 and #2 (with a bin size of 5 μs for a 200-s acquisition time) when driven with shifting voltages of 0.02–0.5 V at the resonant frequency. **b**, **d** The respective FFT amplitude of single molecules #1 and #2 as functions of the driving voltage. Although a small deviation from a linear fit occurs in molecule #2, as a whole, the FFT peaks in both molecules are linearly proportional to the driving voltage.

Supplementary Note 7: CALCULATION FOR EXPERIMENTAL DETECTION SENSITIVITY

Supplementary Figure 7a shows a fluorescence image of a single CV molecule embedded in the PMMA film on the tuning fork when the tuning fork was driven at its resonant frequency of 32.710 kHz. We collected the fluorescence intensity traces by time-correlated single-photon counting when the tuning fork is driven by varying voltages. Since the detection sensitivity strongly depends on the fluorescence intensity, we chose the brightest individual molecules to estimate the highest detection

sensitivity under our experimental conditions. The fluorescence intensity traces with a bin size of 5 μs from an acquisition time of 200 s are shown in Supplementary Figure 7b. We can't directly observe periodic variations of the fluorescence intensity due to the low intensity level of a single molecule with a short bin size of 5 μs. Hence, we performed a fast Fourier transform (FFT) of the fluorescence intensity traces as shown in Supplementary Figure 7c. Obviously, different amplitudes of FFT peak signals are emerging, which corresponds to different driving voltages applied on the tuning fork, and this peak scales linearly with the driving voltage as shown in the inset of Supplementary Figure 7c, which is related to the variations in the amplitude of the fluorescence intensity. The FFT peak position stands for the resonant frequency of the tuning fork or driving frequency generated by a signal generator. The signal remains lower than noise until the driving voltage is 0.05 V. Therefore, we deduce a limit voltage of 0.1 V for the minimum detectable signal with an integration time of 200 s. By identical calculation as in the Main text, we obtained a single-molecule detection sensitivity of 20 pm $Hz^{-1/2}$ for this single molecule.

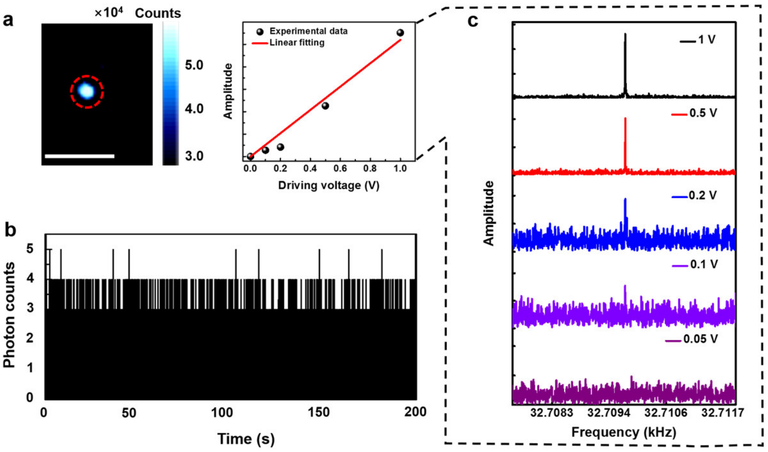

Supplementary Figure 7: Extraction of experimental detection signals for acoustic vibrations. a Fluorescence image of a single CV molecule; Scale bar: 5 μm. **b** Fluorescence photon counts collected by APD (with a bin size of 5 μs for 200-s acquisition time) from a single CV molecule (as shown in **a**) at a driving voltage of 0.2 V. **c** FFT of the fluorescence intensity traces when driven with sinusoidal voltages ranging from 0.05 to 1.0 V at a resonant frequency of 32.710 kHz for the tuning fork. The curves are shifted along the Y axis for clarity. The obvious sharp peak at 32.710 kHz indicates the perfect modulation with the tuning fork and perfect purity of the signal generator. The signal becomes comparable to noise at a driving voltage of 0.05 V, so the limit voltage for a detectable signal is 0.1 V. Inset: The linear dependence of the FFT amplitude as a function of the driving voltage. The fluorescence intensity was kept constant (~15,000 counts s^{-1}) for different driving voltages. The size of a CV molecule is about 2 nm.

Supplementary Note 8: STATISTICAL DISTRIBUTION OF EXPERIMENTAL DETECTION SENSITIVITY

To estimate the detection sensitivity of acoustic waves by single molecules, we collected 283 molecules in total at the resonant frequency of the tuning fork with a driving voltage of 5 V. As a result, we found that there are 81 molecules (29%) with responses to the vibrations of the acoustic wave. Besides, we analyzed statistical distributions of experimental detection sensitivity for different molecules (24 molecules) as shown in Supplementary Figure 8a. Obviously, the sensitivity varies largely. The highest and average detection sensitivity is 10 pm Hz$^{-1/2}$ with ratio of 13% and 200–500 pm Hz$^{-1/2}$ with ratio of 50%, respectively. The large variation of the sensitivity is probably due to the random arrangement of the nanorods and molecules and their orientations respective to each other and relative to the acoustic wave. Then, we also estimated the probability to find the molecule as function of the molecule-nanorod distance among all the detected molecules. To do this, we assume that the molecule which locates at distance shorter than 10 nm can be detected meaning the probability to be 100% at 10 nm. As shown in Supplementary Figure 8b, only 13% of the detected molecules locate close to the nanorod with distance shorter than 2 nm providing high detection sensitivity of 10 pm Hz$^{-1/2}$. Actually, the probability is even lower if we consider that only the molecule which locates close to the tip of the nanorod can provide high sensitivity.

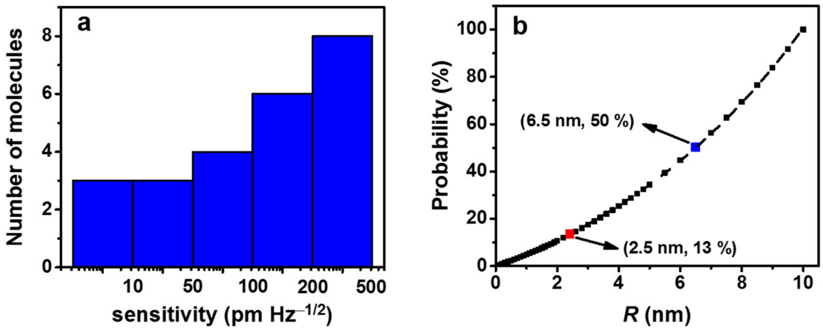

Supplementary Figure 8: Statistical distributions of experimental detection sensitivity. a Histograms of measured detection sensitivities for 24 molecules. The highest detection sensitivity is 10 pm Hz$^{-1/2}$ and the lowest detection sensitivity is 500 pm Hz$^{-1/2}$. **b** The probability to find a molecule as a function of molecule-nanorod distance among all the detected molecules (assuming all the molecules with distance less than 10 nm can be detected). The solid arrows are guide for the eyes.

Supplementary Note 9: THE DETECTION BANDWIDTH OF SINGLE-MOLECULE DETECTOR

The detection bandwidth is an important parameter for an acoustic detector. The detection of the acoustic wave by our single molecule is based on the fluorescence variation which is extracted by FFT analysis of the fluorescence intensity trace. The bandwidth is thus limited by the time resolution (bin size) of the fluorescence intensity trace. In this work, the fluorescence intensity is recorded with time resolution of 4 ps based on a time-correlated single-photon counting (TCSPC) instrument from PicoQuant. Thus, the minimal bin size of 4 ps determines a detection bandwidth of 40 GHz when taking 6 points per oscillation into consideration, according to Nyquist's criterion. Of course, such a high sampling rate would increase the amount of data to process considerably. To prove that the detection sensitivity will not be affected by the bin size, we compared the FFT signal of the same intensity trace with different bin sizes. As shown in Supplementary Figure 9, the signal-to-noise of the FFT signal remains constant when the bin size is decreased down to 1 ns, corresponding to bandwidth of 0.2 GHz. Thus, the detection bandwidth of the single-molecule acoustic detector is DC to 40 GHz, which can be further improved by a single-photon counting instrument with high time resolution. It is worth noting that to achieve such broad bandwidth, we need to excite the molecule many times to collect enough photons, which limits our method's time resolution for real-time measurements. The bandwidth of the real-time measurements is limited by the fluorescence rate of the molecule which is about 4 kHz for the current system. Molecules with shorter lifetime or better optimization could provide a broader bandwidth.

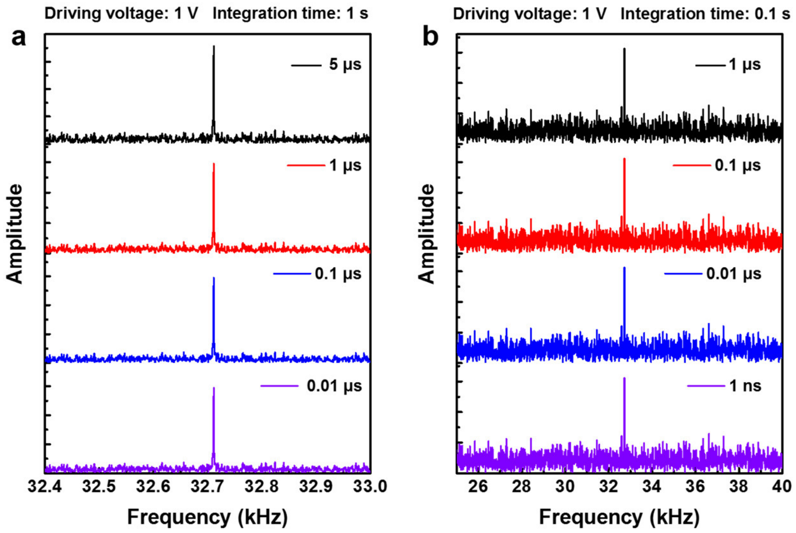

Supplementary Figure 9: FFT of the fluorescence intensity traces with variable bin sizes when driven with a voltage of 1 V at variable integration times. a At an integration time of 1 s. **b** At an integration time of 0.1 s. It is clear that the FFT peaks are completely the same and the signal-to-noise has no variations upon decreasing the bin size. The bandwidth of such a nano-acoustic detector can enable detection of high-frequency acoustic waves up to dozens of gigahertz (GHz).

Supplementary Note 10: NUMERICAL CALCULATION FOR ENHANCEMENT FACTOR

To theoretically calculate the detection sensitivity for acoustic waves with a single molecule, we should first determine the total fluorescence enhancement (F_{tot}) of a single molecule near a gold nanorod, which mainly comes from two contributions, from excitation enhancement (F_{exc}) and from emission enhancement (F_{em}). For dyes with very low fluorescence quantum yield (η<<1), the calculation can be simplified into the following Supplementary Equation (1)[1]:

$$F_{tot} \approx F_{exc} \times F_{em} \qquad (1)$$

F_{exc} is purely electromagnetic and depends only on the excitation wavelength, which is due to improvement in the absorption cross section. The emission enhancement F_{em}, however, involves several optical processes due to improvement in the emission rate, including the radiative decay rate (with enhancement F_{rad}), and the nonradiative decay rate (which adds a contribution K_{nr} to the intrinsic rate k_{nr}^{0} of an isolated molecule). For weak enough excitation, we can calculate F_{em} according to

the following Supplementary Equation (2) [1]:

$$F_{em} = F_{rad} \cdot \frac{k_{nr}^0 + k_r^0}{k_{nr}^0 + K_{nr} + F_{rad} k_r^0} \qquad (2)$$

Where k_r^0 (k_{nr}^0) is the intrinsic radiative (nonradiative) decay rate of the dye, respectively. For a single CV molecule, according to the quantum yield (QY) of 0.5% and fluorescence lifetime of 0.86 ns measured in the PMMA film, k_r^0 and k_{nr}^0 is calculated to be ~6×10^6 and 1.2×10^9 s^{-1}, respectively.

A classical electrodynamics description is usually used to model the molecule as a radiating point-like dipole in the vicinity of a metal surface to theoretically calculate F_{exc}, F_{rad} and K_{nr} because these parameters are purely electromagnetic. Generally speaking, there are several methods such as finite difference time domain (FDTD), discrete dipole approximation (DDA) and finite-element method (FEM) to solve Maxwell's equations to obtain these parameters.

In this work, both F_{exc} and F_{em} were numerically calculated based on a finite-element method (FEM) using Comsol Multiphysics 5.4. The single gold nanorod was modeled as a 3D spherically capped cylinder with dimensions of 25×12×12 nm to produce a longitudinal surface plasmon resonance (SPR, approximately 644 nm in PMMA medium with refractive index of 1.5) close to the value 640 nm measured in a suspension of gold nanorods in DMSO. In this model, the gold nanorod was surrounded by a PMMA layer and a perfectly matched layer (PML) in PMMA medium. The PMMA layer interacted with the incident light to produce the local electric field. The perfectly matched layer was used as the absorption boundary condition to reach complete light absorption. Tetrahedron volume elements with the minimum size of 0.1 nm were used to model the gold nanorod in order to approach the real solution as precisely as possible. In addition, the refractive constants of the gold nanorod were taken from Johnson and Christy[2], and the refractive index in PMMA medium was set as 1.5. The scattered-field pattern was used to simulate the localized electric field of the gold nanorod excited with linearly polarized light of 633 nm to calculate F_{exc}. Ideally, the wave vector direction of the incident light was set to

be perpendicular to the longitudinal axis of the nanorod and the orientation of the transition dipole moment of the CV molecule was set parallel to the long axis of the gold nanorod as shown in inset of Fig. 3a in the main text, at a variable distance from the metal surface. F_{exc} as a function of the molecule-distance was calculated from the square ratio between the localized field intensity and the incident field intensity, according to the following Supplementary Equation (3)[3]

$$F_{exc} = |\mathbf{E}|^2 / |\mathbf{E}_0|^2 \quad (3)$$

where $\mathbf{E}$ is the local field intensity and $\mathbf{E}_0$ is the incident field intensity.

We used a radiating point-like dipole with identical emission characteristics of the CV molecule as the radiating source in the full-field pattern to calculate F_{em} while all the other conditions were kept the same as for the calculation of F_{exc}. The emission wavelength of the point-like dipole was fixed at the emission peak of 640 nm for CV and the orientation of the molecule was set to be parallel to the longitudinal axis of the nanorod to simulate and calculate the maximum F_{em}. For the calculation of F_{em}, we divided it into four steps. First, we calculated the time-averaged power radiated by an isolated CV molecule without a gold nanorod (P_r) by integrating the average Poynting flux over the PMMA layer surface. Second, we calculated the time-averaged power radiated by a CV molecule with a gold nanorod (P_{rad}) by integrating the average Poynting flux over the PMMA layer surface. Third, we calculated the time-averaged power absorbed by the gold nanorod (P_{abs}) due to Ohmic loss. Finally, we calculated the radiative decay rate enhancement (F_{rad}) and the additional nonradiative decay rate (K_{nr}) using the following Supplementary Equations (4)[4,5]:

$$F_{rad} = k_r / k_r^0 = P_{rad} / P_r \qquad K_{nr} / k_r^0 = P_{abs} / P_r \quad (4)$$

Hence, we can calculate the maximum F_{em} using Supplementary Equation (2) and the maximum F_{tot} using Supplementary Equation (1) mentioned above.

Supplementary Note 11: CALCULATION OF RADIATIVE ENHANCEMENT AND ADDITIONAL NONRADIATIVE DECAY RATE

As shown in Supplementary Figure 10, we calculated the radiative decay rate enhancement (F_{rad}) and the additional nonradiative decay rate (K_{nr}) according to the Supplementary Equation (4) as mentioned above. F_{rad} monotonically increases as the distance decreases until a minimum distance of 0.5 nm is reached**.** At shorter distances, however, the fluorescence quenching becomes dominant, and F_{rad} decreases again. K_{nr} increases exponentially with decreasing the distance, but it increases more sharply than F_{rad}.

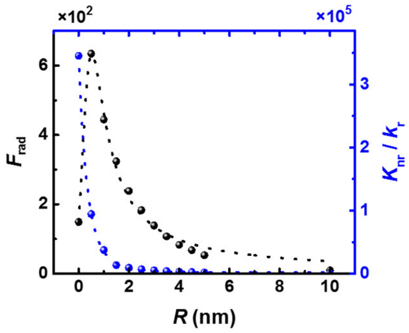

Supplementary Figure 10: Calculation of the radiative decay rate enhancement (F_{rad}) and the additional nonradiative decay rate (K_{nr}) as functions of the molecule-tip distance (R). F_{rad} increases sharply when the molecule-tip distance is less than 0.5 nm and decreases exponentially when the distance is greater than 0.5 nm. K_{nr} decays exponentially as a function of distance, but it decreases more sharply than F_{rad}.

Supplementary Note 12: CALCULATION OF WAVELENGTH DEPENDENCE OF ENHANCEMENT

We calculated the radiative decay rate enhancement (F_{rad}) and the additional nonradiative decay rate (K_{nr}) as functions of the emission wavelength for the point-like dipole of a CV molecule located at a fixed molecule-tip distance of 5 nm. As can be seen from Supplementary Figures 11a and 11b, both F_{rad} (red spheres) and K_{nr} (blue spheres) strongly depend on the emission wavelength and show a good agreement with the extinction spectrum (black spheres) of the gold nanorod, although there is a slight red shift of the peak with 650 nm (~6 nm compared to the SPR of 644 nm of the gold nanorod in PMMA media) and asymmetric line shape for wavelength

dependence of F_{rad} compared to the extinction spectrum of the gold nanorod, which was also reported by other researchers[1]. These slight differences mainly come from the damping of the nanorod[6], the dephasing of the near field[7] and the phase difference between the molecule's dipole and the driven SPR dipole[1].

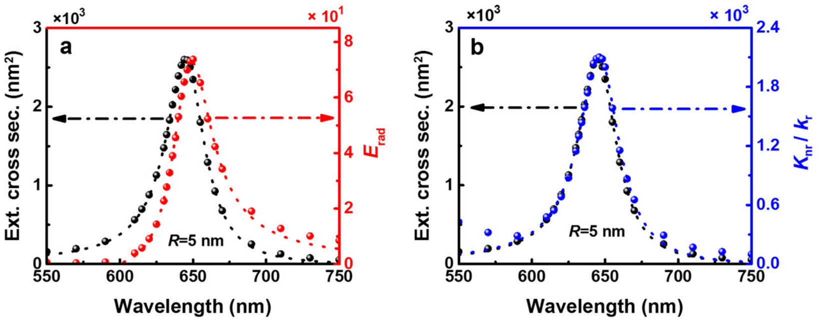

Supplementary Figure 11: Calculated spectra of the extinction coefficient of the gold nanorod, its radiative enhancement factor F_{rad} as well as its additional nonradiative relaxation rate K_{nr} as functions of the emission wavelength of a CV molecule. a Calculated radiative decay rate enhancement factor (F_{rad}, red spheres) as a function of emission wavelength of the point-like dipole of the CV molecule. F_{rad} strongly depends on the emission wavelength and shows a good agreement with the extinction spectrum of the gold nanorod, although there is a slight red shift in the peak with 650 nm (~6 nm compared to the SPR of 644 nm of the gold nanorod) and an asymmetric line shape. **b** The additional nonradiative decay rate (K_{nr}, blue spheres) as a function of emission wavelength of the point-like dipole of the CV molecule. K_{nr} depends on the emission wavelength and is consistent with the extinction spectrum of the gold nanorod. The extinction spectrum of the nanorod as a function of the incident wavelength is shown by the black spheres in **a** and **b**. The distance between the molecule and the gold nanorod was fixed at 5 nm. The dotted lines are the fitting curves of the scattered points.

Supplementary Note 13: CALCULATION OF THE DETECTION SENSITIVITY LIMIT

The theoretical detection sensitivity of the acoustic wave is determined by the smallest detectable deformation over an acquisition time of t against the shot noise associated with the fluorescence intensity traces, according to Supplementary Equation (5) [8]:

$$\delta I \cdot t = \sqrt{I \cdot t} \qquad (5)$$

Where I is the fluorescence intensity, t is the acquisition time controlled by time-tagged single-photon counting and δI is the fluorescence intensity variation of I during t. The variation of F_{tot} as a function of the molecule-tip distance is given

by the following Supplementary Equation (6):

$$\delta F_{\mathrm{tot}} = \delta R_{\mathrm{min}} \cdot (\mathrm{d}F_{\mathrm{tot}} / \mathrm{d}R)_{\mathrm{max}} \quad (6)$$

where δR_{min} is the minimum variation of the molecule-tip distance for a detectable signal against the shot noise, i.e., the detection sensitivity, $(\mathrm{d}F_{\mathrm{tot}} / \mathrm{d}R)_{\mathrm{max}}$ is the largest value of the first derivative curves of the fitted F_{tot} as a function of the molecule-tip distance and δF_{tot} is the minimum variation of F_{tot} at given δR_{min}. In this case, δF_{tot} leads to δI, which can be calculated according to the following Supplementary Equation (7):

$$\delta I = I \cdot \delta F_{\mathrm{tot}} \quad (7)$$

Finally, we can obtain the whole calculation for the theoretical detection sensitivity of the acoustic wave, which is given by the Supplementary Equation (8):

$$\delta R_{\mathrm{min}} = (\mathrm{d}R / \mathrm{d}F_{\mathrm{tot}}) / \sqrt{I \cdot t} \quad (8)$$

As a result, at room temperature we could achieve the theoretical detection sensitivity of 10 fm $\mathrm{Hz}^{-1/2}$ for acoustic waves generated by the tuning fork with a single molecule by applying $t = 1$ s, $I = 26{,}000$ counts s^{-1}, $(\mathrm{d}F_{\mathrm{tot}} / \mathrm{d}R)_{\mathrm{max}} = 700$ nm^{-1} into the Supplementary Equation (8), which is 3 orders of magnitude higher than our best experimental detection sensitivity of 10 pm $\mathrm{Hz}^{-1/2}$. We attribute the discrepancy to the following two reasons. On the one hand, we estimated the theoretical maximum sensitivity at the maximum of the first derivative of the total fluorescence enhancement, which is an optimized molecule-nanorod distance. As the molecule-tip distance and the molecular orientation are absolutely random, it is almost impossible to reach the optimized condition for both the molecule-tip distance and the fluorescence enhancement factor in the current version of our experiment. According to estimations described above, the most sensitive molecules in practical experiments are located at a molecule-nanorod distance of 2–3 nm, which is still far away from the best theoretical molecule-nanorod distance of 0.7 nm. To improve the detection sensitivity, position and orientation of the molecule and nanorod have to be controlled, for example by DNA binding[9]. On the other hand, other experimental conditions

could also affect the detection sensitivity, including effects from environmental noise, the collection efficiency of the fluorescence photons, the detection efficiency of the photodiode and the possible blinking behavior of the molecule. Consequently, it is reasonable that the detection sensitivity under our experimental conditions should be much lower than the theoretical optimum.

Supplementary Note 14: EXCLUSION OF FLUORESCENCE VARIATION INDUCED BY THE ELECTRIC FIELD

The fluorescence intensity of a single molecule is very sensitive to the surrounding environment, including the applied electric field. Thus the applied periodic voltage could possibly induce periodic fluorescence intensity variations. It is more convincing that we should measure the Stark effect from the driving voltage applied to the molecule without the gold nanorod around. However, we were unable to do this measurement due to the very weak fluorescence signals (Supplementary Figure 12b) limited by the low quantum yield without fluorescence enhancement compared to the bright spot enhanced by the gold nanorod around (Supplementary Figure 12a). Thus, to confirm that the fluorescence intensity variation comes from acoustic waves generated by the tuning fork instead of the electric field, we investigated the same molecules at a fixed driving voltage with different driving frequencies ranging from 22.732 to 37.732 kHz with an interval frequency of 5 kHz (The resonant frequency of this sample is 32.732 kHz). As can be seen from the molecule #1 and molecule #2 in Supplementary Figures 12c and 12d, there is no signal at off-resonant frequencies of the tuning fork until reaching a resonant frequency of 32.732 kHz. Therefore, we can directly exclude the fluorescence intensity variation coming from the electric field. Otherwise, a similar signal can be observed independently of the driving frequency. In addition, it is previously reported that the external electric field can induce the Stark effect of the fluorescent molecules (about 1 GHz for 1.5 MV/cm)[10]. However, the applied electric field in our experiment is 15 V/cm which is very small and can be neglected, even with resonant enhancement.

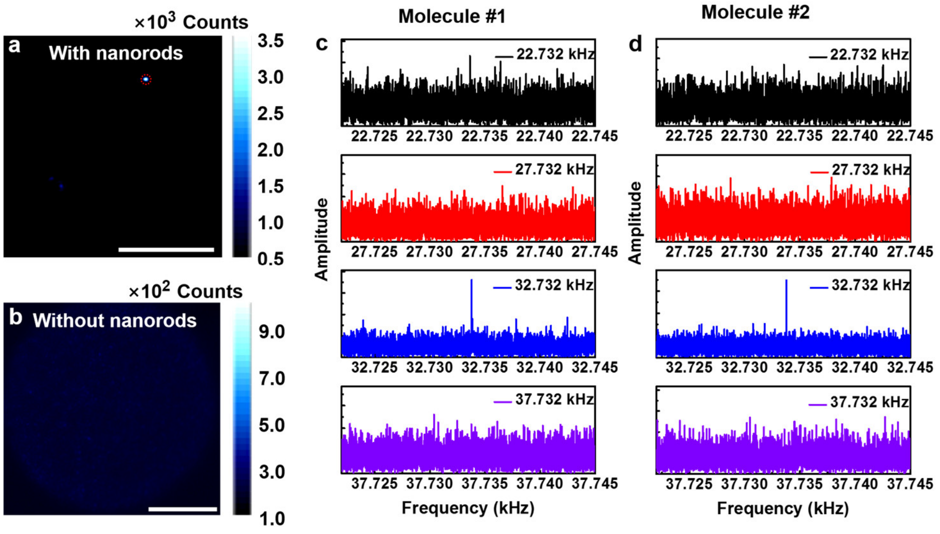

Supplementary Figure 12: Exclusion of the fluorescence intensity variation from the electric field. a Fluorescence image of CV molecules with gold nanorods. The bright spot is enhanced by the gold nanorod around; Scale bar: 20 μm. **b** Fluorescence image of CV molecules without gold nanorods; Scale bar: 20 μm. The intensity is comparable to the background due to the very low quantum yield. **c** FFT amplitude of fluorescence intensity variations of Molecule #1 for a few different driving frequencies and a fixed driving voltage of 2 V. **d** Similar FFT spectrum of fluorescence intensity variations of Molecule #2 at a fixed driving voltage of 0.5 V. There is no signal detected when the driving frequency is off-resonance of the tuning fork, which directly excludes the effect of the electric field.

Supplementary Note 15: EXCLUSION OF FLUORESCENCE VARIATION INDUCED BY MOLECULE DISPLACEMENT

Since the tuning fork vibrates periodically, it is possible that the fluorescence intensity variation could be induced by displacement of the molecule. We can exclude this effect from two aspects: 1) actually, the displacement of the tuning fork arms is very small (~20 nm) under our experimental conditions. The displacement of the molecules located precisely in the middle between the two arms should be at most about one order of magnitude smaller (~2 nm), which is far below the diffraction limit. The fluorescence intensity variation caused by such small displacement is negligible especially when the sample is excited under a wide-field microscope. 2) We found that even two molecules in the same field of view show completely different behaviors. In the same field of view (Supplementary Figure 13a) with absolutely identical experiment conditions, we found that a single molecule #1 shows significant fluorescence intensity variation (high amplitude of FFT signal) while there is hardly

any fluorescence variation observed for a single molecule #2 (Supplementary Figure 13b). If the fluorescence intensity variation was caused by the displacement of the molecules, we would expect similar results for these two molecules. The reason for this difference is that these two molecule-nanorod systems have different orientations. The molecule can sense the acoustic wave only when the strain caused by the acoustic wave has a component along the longitudinal axis of the gold nanorod showing fluorescence variation. When the acoustic wave has no strain component along the longitudinal axis of the gold nanorod, the fluorescence of the molecule will remain constant because the distance between the molecule and the nanorod does not change.

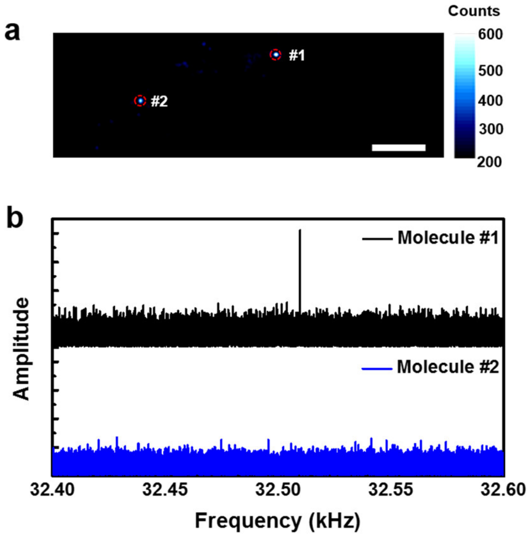

Supplementary Figure 13: Exclusion of the fluorescence intensity variation from molecule displacement. a A fluorescence image of two single CV molecules from the same field of view; Scale bar: 10 μm. **b** Fast Fourier transform (FFT) of the fluorescence intensity traces of these two molecules (200-s acquisition time). The tuning fork was driven with a fixed voltage of 3 V at a resonant frequency of 32.508 kHz. These two single molecule/nanorod systems are highlighted by the red dashed circles for clarity.

Supplementary Note 16: EXCLUSION OF EFFECT INDUCED BY THE DENSITY VARIATION OF THE MATRIX

The plasmonic effect is very sensitive to the refractive index of the medium. The oscillation of the tuning fork also induces variation of the film density which can alter the refractive index around the gold nanorod. In this case, the localized electric field will change or the resonant spectrum will be shifted causing fluorescence intensity variation. Under our experimental conditions, the relative deformation of the film is

calculated to be ~7×10^{-5}, which causes the maximum density change of 7×10^{-5} assuming that the thickness and width of the film remained unchanged. Thus, the relative variation of the refractive index of the film is approximately 4×10^{-5} based on the Clausius-Mossotti relation ($\frac{\Delta n}{n-1} = \frac{\Delta \rho}{\rho}$)[11]. Then, we estimated the change of the total enhancement factor (F_{tot}) when the refractive index was changed from 1.50 into 1.49 by Comsol Multiphysics 5.4 software. As shown in Table 1, the largest change of F_{tot} is 16% when the molecule-tip distance is 1.5 nm. Thus, we can obtain the relative variation of 9×10^{-4} of F_{tot} for the refractive index change of 4×10^{-5}. Then, the maximum fluorescence intensity variation can be calculated to be ~20 counts when taking the fluorescence intensity of 26,000 counts s^{-1} into calculation. For comparison, we calculated the fluorescence intensity variation from the molecule-nanorod distance variation caused by the acoustic strain. The minimum molecule-nanorod distance variation in our experiments is 0.7 pm and the biggest variation of the total fluorescence enhancement factor as a function of the molecule-nanorod distance is calculated to be ~700 nm^{-1}, i.e., $(\mathrm{d}F_{\text{tot}} / \mathrm{d}R)_{\max} = 700\ \text{nm}^{-1}$ as mentioned below. Then, we can obtain a total fluorescence enhancement factor variation of 0.49. Finally, the fluorescence intensity variation can be calculated to be ~13,000 counts when taking the fluorescence intensity of 26,000 counts s^{-1} into calculation, which is about 3 orders of magnitude higher than that of the density variation. As a result, we can directly exclude the density variation effect on the response to the strain caused by the acoustic wave.

Supplementary Table 1: Dependence of F_{tot} at different fixed molecule-tip distances on the changes of the refractive index in the medium.

Refractive index \ Distance (nm)	1.0	1.5	2.0
1.50	620	876	684
1.49	736	1016	796

Supplementary Note 17: EXCLUSION OF EFFECT FROM THE GOLD NANOROD'S PHOTOLUMINESCENCE

Besides the fluorescence of the molecule, the photoluminescence of the gold nanorod can also be possibly detected although the quantum yield is extremely low. The acoustic wave may directly induce deformation of the nanorod causing variation of its photoluminescence. Therefore, in order to exclude the influence of the gold nanorod's photoluminescence, we have measured the photoluminescence of gold nanorods embedded in the PMMA film without CV molecules and their corresponding response to acoustic waves. As shown in the two representative gold nanorods in Supplementary Figure 14, we found that there are no obvious FFT signals present when the tuning fork was driven at its resonant frequency of 32.718 kHz under a driving voltage of 5 V. In addition, the fluorescence rate of a single CV molecule is ~26,000 counts s^{-1} at an excitation of 50 W cm^{-2} due to the large fluorescence enhancement effect of the nearby gold nanorod. The fluorescence rate of a single gold nanorod is ~30 counts s^{-1} at an excitation of 500 W cm^{-2} due to very low PL quantum yield (10^{-5}–10^{-6})[12]. Thus, the photoluminescence intensity of the gold nanorod is about 3–4 orders of magnitude lower than the fluorescence of an enhanced CV molecule close to a gold nanorod. Therefore, we can completely exclude the effect from the photoluminescence of the gold nanorod.

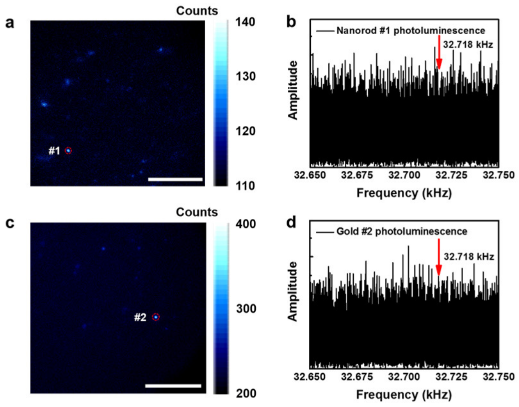

Supplementary Figure 14: Exclusion of the obtained signal from the photoluminescence variation of the gold nanorod. a, **c** Photoluminescence images of nanorod #1 and #2, respectively; Scale bar: 20 μm. **b**, **d** The responses of nanorod #1 and #2 to the acoustic wave, respectively. Both of two gold nanorods have no responses to the acoustic wave. The tuning fork was driven at its resonant frequency of 32.718 kHz under a driving voltage of 5 V. The resonant frequency of the tuning fork is highlighted by the red solid arrow for the eyes.

Supplementary Note 18: EXCLUSION OF THE MOLECULAR STRUCTURE DEFORMATION

In principle, the structure of the CV molecule is much rigid due to the strong covalent bonding compared to the distance change based on the weak inter-molecule interaction. It is difficult for the weak acoustic strains to induce a conformational change of a rigid CV molecule. Thus, we think it is a fair assumption to neglect changes of molecular conformation for such small displacements. Moreover, as our detection frequency is much lower than the vibration frequencies of chemical bonds, coupling between them can be safely neglected.

To exclude any possible effect from the conformation change on the acoustic detection, we checked the fluorescence variations of a PMMA film doped with a high concentration CV molecules without gold nanorods during the oscillation of tuning fork. As shown in Supplementary Figure 15, we did not observe any signal from the FFT analysis. These results directly exclude the possible effect from deformation of the molecular structure.

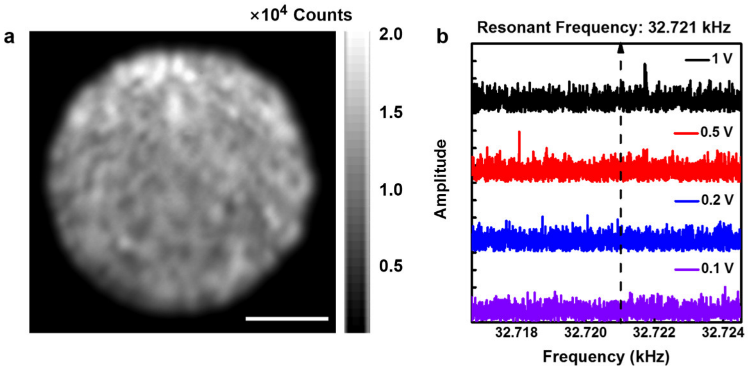

Supplementary Figure 15: Exclusion of the molecular structure deformation. a Fluorescence image of a PMMA film with a high CV concentration; Scale bar: 20 μm. **b** FFT of the fluorescence intensity traces of CV films driven with voltages ranging from 0.1 to 1.0 V applied at the resonant frequency of the tuning fork.

Supplementary Note 19: RESPONSE OF THE GOLD NANOROD'S SCATTERING INTENSITY TO THE ACOUSTIC WAVE

The strain caused by the acoustic wave generated by the tuning fork might modulate the plasmon's resonance of the gold nanorod by either changing the refractive index of the film or inducing a deformation of the gold nanorod, which can both change the

localized electric field and induce the spectrum shift. Therefore, we investigated the scattering light of a single gold nanorod embedded in the PMMA film without CV molecules and the corresponding response to the strain caused by the acoustic wave. In order to compare with single-molecule fluorescence response to the acoustic strain, we controlled the scattering intensity of a gold nanorod to be ~48,000 counts s^{-1} which is close to the fluorescence intensity of a single CV molecule with 26,000 counts s^{-1}. Supplementary Figure 16a shows a scattering image of a single gold nanorod when the tuning fork was driven at its resonant frequency of 32.719 kHz. The target gold nanorod for the acoustic wave detection is highlighted by the red dotted circle. We collected the scattering intensity traces by time-correlated single-photon counting when the tuning fork is driven by varying voltages. Similarly, we also performed a fast Fourier transform (FFT) of the scattering intensity traces with a bin size of 5 μs from an acquisition time of 200 s as shown in Supplementary Figure 16b. Obviously, different amplitudes of FFT peak signals are emerging, which corresponds to different driving voltages applied on the tuning fork, and this peak scales linearly with the driving voltage, which is related to the variations in the amplitude of the scattering intensity. The FFT peak position stands for the resonant frequency of the tuning fork or driving frequency generated by the signal generator. The signal becomes comparable to the noise at the driving voltage of 0.2 V. Therefore, we deduce a limit voltage of 0.5 V for the minimum detectable signal with an integration time of 200 s. By identical calculation as in the Main text, we obtained a single-nanorod detection sensitivity of 100 pm $Hz^{-1/2}$ for this single gold nanorod. This detection sensitivity is one order of magnitude lower than that of a single-molecule fluorescence based on nanorod molecule distance variation caused by the acoustic strain, which indicates the great superiority of the single-molecule fluorescence detection method. In both cases, however, the molecule-nanorod system can act as a nano detector of acoustic deformations.

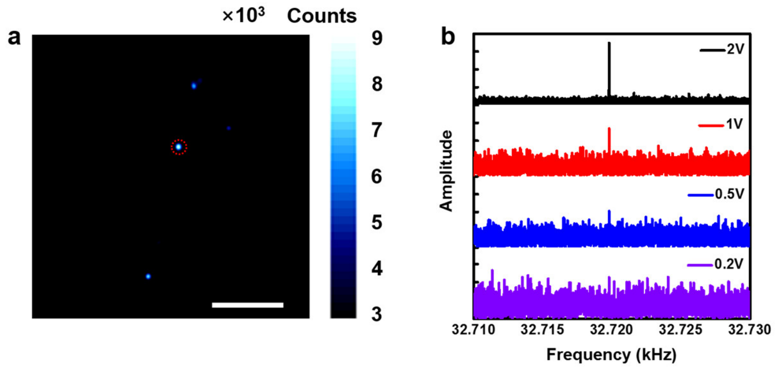

Supplementary Figure 16: Response of the gold nanorod's scattering intensity variation to the acoustic wave. a A scattering image of gold nanorods , the gold nanorod highlighted by the red dotted circle was measured to see the response to the acoustic wave; Scale bar: 10 μm. **b** Fast Fourier transform (FFT) of the scattering intensity traces (with a bin size of 5 μs for a 200-s acquisition time) when driven with sinusoidal voltages ranging from 0.2 to 2.0 V at a resonant frequency of 32.719 kHz for the tuning fork. The curves are shifted along the Y axis for clarity. The obvious sharp peak at 32.719 kHz indicates the perfect modulation with the tuning fork and perfect purity of the signal generator. The signal becomes comparable to noise at a driving voltage of 0.2 V, so the limit voltage for a detectable signal is 0.5 V. The scattering intensity was kept constant (~48,000 counts s^{-1}) for different driving voltages.

Supplementary References

1. Khatua, S., Paulo, P. M. R., Yuan, H., Gupta, A., Zijlstra, P. & Orrit, M. Resonant plasmonic enhancement of single-molecule fluorescence by individual gold nanorods. *ACS Nano* **8**, 4440–4449 (2014).
2. Johnson, P. B. & Christy, R. W. Optical constants of the noble metals. *Phys. Rev. B* **6**, 4370–4379 (1972).
3. Zijlstra, P., Paulo, P. M. R. & Orrit, M. Optical detection of single non-absorbing molecules using the surface plasmon resonance of a gold nanorod. *Nat. Nanotechnol.* **7**, 379–382 (2012).
4. Meng, X., Grote, R. R., Dadap, J. I., Panoiu, N. C. & Osgood, R. M. Engineering metal-nanoantennae/dye complexes for maximum fluorescence enhancement. *Opt. Express* **22**, 22018–22030 (2014).
5. Zhang, W., Caldarola, M., Lu, X. & Orrit, M. Plasmonic enhancement of two-photon-excited luminescence of single quantum dots by individual gold nanorods. *ACS Photonics* **5**, 2960–2968 (2018).
6. Zuloaga, J. & Nordlander, P. On the energy shift between near-field and

far-field peak intensities in localized plasmon systems. *Nano Lett.* **11**, 1280–1283 (2011).

7. Aizpura, J. & Esteban, R. *Optical antennas* Ch.1 (Cambridge Univ. Press, Cambridge, 2013).
8. Zmuidzinas, J. On the use of shot noise for photon counting. *Astrophys. J.* **813**, 17 (2015).
9. W. Zhang, M. Caldarola, X. Lu, B. Pradhan and M. Orrit, Single-molecule fluorescence enhancement of a near-infrared dye by gold nanorods using DNA transient binding, *Phys. Chem. Chem. Phys.*, 2018, **20**, 20468–20475.
10. Moradi, A., Ristanović, Z., Orrit, M., Deperasińska, I. & Kozankiewicz, B. Matrix-induced linear Stark effect of single dibenzoterrylene molecules in 2,3-dibromonaphthalene crystal. *ChemPhysChem* **20**, 55–61 (2019).
11. Talebian, E. & Talebian, M. A general review on the derivation of Clausius-Mossotti relation. *Optik* **124**, 2324–2326 (2013).
12. Yorulmaz, M., Khatua, S., Zijlstra, P., Gaiduk, A. & Orrit, M. Luminescence quantum yield of single gold nanorods. *Nano Lett.* **12**, 4385–4391 (2012).
